# Emerging Health and Education Issues Related to Internet Technologies and Addictive Problems

**DOI:** 10.3390/ijerph18010321

**Published:** 2021-01-04

**Authors:** Olatz Lopez-Fernandez

**Affiliations:** 1 Turning Point, Eastern Health Clinical School, Monash University, 110 Church Street, Richmond, VIC 3121, Australia; Lopez.Olatz@gmail.com or Olatz.Lopez@quironsalud.es; 2 Fundación Jiménez Díaz University Hospital Health Research Institute, Avda. Reyes Católicos 2, 28040 Madrid, Spain

**Keywords:** Internet addiction, Internet problematic use, problematic mobile phone use, Internet use-related addiction problems, technologies, education, health, treatment, prevention, COVID-19

## Abstract

This timely editorial paper outlines some of the main emerging research on technological topics on health and education approaches to Internet use-related problems before and during the beginning of coronavirus disease 2019 (COVID-19). Background: The aim is to provide a brief overview to facilitate a rapid comprehensive and practical approach to these new trends to promote research, interventions, education, and prevention. Methods: The rapid review includes an analysis of both health and education technologies studies on Internet use-related addiction problems included in the Special Issue “Internet and Smartphone Use-Related Addiction Health Problems: Treatment, Education and Research” to extract recent findings and a few reflections about the development of the field before and during the first wave of the COVID-19. Results: Main findings highlighted studies which tended to be empirical, with a relational type associating specific addictive problems with individual and a few contextual factors in adult populations. Psychometric studies about scales are prevalent, but predictive and mixed methods ones are starting to emerge, together with reviews about conceptualisation, measure, treatment, and prevention. Conclusions: From the arrival of Internet, our societies have converged in a global culture which has impacted health and educational domains. Internet use-related addiction problems have globally emerged and common knowledge, advances, and strategies exist to overcome challenges which are starting to be tested, and prevention interest has arisen in a pandemic situation with global health problems holistically tackled.

## 1. Introduction

Over the last 25 years there has globally been an increasing use of Internet technologies and electronic devices in our daily lives, especially during the last two decades according to the International Telecommunication Union (ITU [1]). The Internet, usually defined as a set of Information and Communication Technologies (ICTs), is now a global network which has inter-connected more than half of the world population [1], who are informed, and constantly communicated (e.g., via social media, messaging applications (apps)) through domestic and mobile devices (e.g., smartphone). However, negative aspects have commonly emerged producing health, educational, and social problems, such as intercultural or intergenerational gaps (e.g., in African countries there is a dual digital divide [2], and older generations still live the grey digital divide [3]).

Our societies have progressively changed, having become more digital in many ways despite the online inequalities. It has especially impacted our human behaviours because of the ubiquity of the Internet on our everyday life activities [4]. This increase of online uses has had important benefits for health (e.g., eHealth), education (e.g., eLearning), and social life (e.g., social media) sectors, but problems have arisen (e.g., Internet use-related addiction problems, cyberbullying, or cybercrime). Furthermore, the coronavirus disease 2019 (COVID-19) has unexpectedly impacted our societies, strengthening the role of ICT in our new restrictive daily lives to support the rapid public health response worldwide [5].

In the psychological field, the Internet has transformed some of our regular behaviours, which have been enriched due to the new contexts we live in (i.e., online, off-line, and both), and opportunities have emerged (e.g., to working remotely via the Internet from home or any other place, to enjoy streaming TV shows online through laptops, smartphones, etc.). Individuals now manage themselves in online and offline domains indistinctively using many devices and apps. The psychology of the Internet (or Cyberpsychology), therefore, is studying the relationship between ICT and the psychological mechanisms, covering both positive and negative issues in a cyberspace which is digitally and humanistic mediated. According to Wallace [6], for instance, the taxonomy of online environments includes the web, the deep (or dark) web, email, forums, chats, blogs, social networks, texting, and virtual reality, which are almost all usually managed by individuals connected through the Internet, especially through smartphones.

The COVID-19 pandemic has made these environments part of our daily lives and pushed us to use ICT in great parts of our daily routines, work or study duties, and entertainment, and to become a part of our personal and social lives. These online settings are, therefore, influencing our daily emotions, cognitions, and behaviours, such as the case of some excessive online habits which put a small proportion of the population (e.g., youth) at risk of developing addiction problems. Internet addiction (IA) or problematic Internet use is understood as a set of potential addictive online problems [7] (reason why in this paper it is used the term “Internet use-related addiction problems”); but the only formally recognised problem is (video) gaming disorder (GD [8]). However, a broader spectrum of Internet use-related addiction problems have largely been researched beyond GD [9]; mainly through descriptive and relational studies (i.e., studies which look for associations among variables) about social media addiction (SMA; e.g., Facebook addiction), problem mobile phone use (PMPU; e.g., problem smartphone use (PSU)), cybersex addiction (e.g., problematic usage of pornography (PUP)), online gambling (OG), buying addiction (e.g., problem Internet shopping (PIS)), among other related problems (e.g., cyberchondria).

Reasons which drive the generation of this second Special Issue “Internet and Smartphone Use-Related Addiction Health Problems: Treatment, Education and Research” after the successful previous Special Issue “Internet and Mobile Phone Addiction: Health and Educational Effects” [10]. The present introductory article offers a rapid overview of the new advances on Internet use-related addiction problems collected from health and education approaches before and during COVID-19, and to offer a few editorial reflections from environmental and cyberpsychology perspectives.

## 2. Materials and Methods

This study is a rapid review (i.e., rapid evidence assessment), an evidence-based knowledge synthesis. The systematic review process components are simplified to produce information as a summary of the evidence discovered promptly. The aim was to examine articles included in the cited Special Issue (2019–2020), which were qualitatively reviewed by the author (i.e., invited editor) through a content analysis to provide a rapid outline of the state of the art on these addictive problems associated with using current technologies from a health, educational, or both settings simultaneously. The procedure was the following: (i) to collect all published papers within the Special Issue, (ii) to design a set of variables in a table to rapidly review them one by one (i.e., see Results section and Table 1), (iii) to read article by article extracting all the information previously included in the table (i.e., columns), and (iv) to write the information extracted in the table.

The methodological aim of this type of research study is to collect evidence-based conclusion to rapidly make an informed decision usually by policymakers or to justify the need for further research. In the present editorial paper, the pretension has been to inform within the same year of closing the Special Issue, about how this productive field of Internet-use related addiction problems has grown in pre-COVID times and how it seems the pandemic has also swift the productivity and priorities of this clinical and scientific field.

## 3. Results

The main content of the articles is presented in Table 1. It includes the following set of variables to analyse the characteristics of the Special Issue:

(a) The authors’ names, country where these authors are affiliated, and continent(s);
(b) The setting in which data were collected: if data were collected at an educational institution (e.g., in a secondary school or a university) the setting is labelled as ‘Education’, if they were collected through a non-educative website (e.g., through a social network) the setting is labelled ‘Social’, while if data have been collected in a health setting (e.g., a hospital or health centre with patients), the setting is labelled ‘Health’, and the sector in which the study can be situated (i.e., ‘Health’ if the main aim is to contribute to health sciences regarding these problems; or ‘Education’ if the primary aim is to impact educational facets of the problem);
(c) The main method (i.e., empirical-quantitative, qualitative, or mixed method; or theoretical study-review, theoretical model, etc.), type of study (i.e., descriptive study, if the paper only provides detailed definitions and observations of the characteristics of the phenomenon under study; psychometric study—if the aim of the study is testing the psychometric properties of a scale, questionnaire or test assessing Internet use-related addiction problems, such as the reliability and validity to create or adapt a psychometric tool for a specific language or cultural group; relational study, or correlational study, which refers to the aim of associating a set of characteristics of the phenomenon without predictive aims; and predictive study, which implies a statistical or manipulative design to treat a set of variables as predictors or causes of another set of variables, the outcomes or dependent variables), population groups (e.g., children, adolescents, adults), and time of data collection (i.e., by years, if included in the paper);
(d) The main addictive online problem(s) researched: e.g., IA, GD, SMA, PMPU, PSU, PUP, OG, and PIS.
(e) The overall main findings of the study.

## 4. Discussion

The present editorial paper offers a rapid overview of the pre-COVID-19 advances on Internet use-related addiction problems research collected in this second special issue. The main aim was to invite research on these health problems from the areas of treatments, education, and other research advances in the field. After the first wave of the pandemic, the editor would also like to share a few reflections from cyberpsychology and environmental perspectives.

Regarding the first main finding, compared with the previous Special Issue on this theme [10], using the same even stricter peer-review process, the second Special Issue has collected double the number of papers (i.e., 40 versus 20 papers) in approximately a year, which indicates the increasing interest and publication rate in this research field. Related to the authors’ affiliations, this second Special Issue includes peer-reviewed papers from international samples (ordered by frequency): Europe (i.e., Spain, Germany, UK, France, Turkey, Sweden, Czech Republic, Slovakia, and Poland), Asia (i.e., China, Korea, Taiwan, Hong Kong, Malaysia, and Iran), America (USA, Canada, Perú, and Colombia), and one from Australia. However, no papers were included from Africa, which is consistent with the existence of the dual digital divide and international data reported [1,2]. This fact does not mean that there is no existence of these type of problems on this continent, but it highlights that no work has been submitted for a review in this case as we were sensitive to it.

On the other hand, the main aim was to cover health and education approaches in the context of these problems, which has partially been achieved, as the majority of papers (74%) included both approaches. However, half of the papers (54%) were studies undertaken in educational settings (i.e., high schools [14,18], universities [12,13]) with a health purpose, such as to adapt a diagnostic tool [12,13,20,21,32,37,45] or to study psychological mechanisms or comorbid problems which affect or mediate these problems to be used in prevention and treatment plans [17,22,23,24,25,46]. Only a few studies (20%) were conducted in health settings (i.e., hospitals [44] or health centres [33]) or were health reviews about these problems [46]). In the current literature, the method of extracting health knowledge through educational environments is quite common, but at the same time it shows its maturity is still compromise, as more research is needed in non-educational and clinical sectors with qualitative, mixed methods and manipulative research designs (e.g., quasi-experiments and experiments) to go in-depth on knowing better what the mechanisms behind these problems are, what seems to be the cause and how it affects to the prognostic. Thus, the need for extracting research on treatments was limited in this issue, which is a challenge the field needs to overcome.

The problems studied are more specific IA problems rather than generalised IA (23% of the papers), which is a new trend on the field, and it is conversely opposite to the first two decades of research, in which IA was more prevalent than specific Internet use-related addiction problems. In order of frequency, in this second Special Issue, which was open to research on all addictive Internet and mobile phone use, the most prevalent problems ordered by frequency were: PMPU (38% [27]), IA (26% [16]), GD (23% [19]), SMA (18% [26]), OG (8% [39]), PUP (5% [37]), and PIS (3% [23]). This means the tendency before COVID-19 in the field was to study addictive problems related to the use of mobile technologies (e.g., PSU) and its risks, and this has increased during the pandemic [50]. Reasons for this include those problems being the most researched topics due to the need of increasing and controlling our safety behaviours while travelling or commuting during the pandemic, in addition to other advantages and continuous innovations offered by mobile technologies (e.g., apps, aids, and trackers). Furthermore, as predicted a few years ago [9], other types of problematic addictive use apart from GD are studied and causing concern in population groups (e.g., OG and PUP).

This is consistent with the methodology used in pre-COVID research on Internet use-related addiction problems papers published in the Special Issue (i.e., which collected data between 2013 and 2019). Almost all works were empirical (90%) with quantitative studies (only two mixed methods and no qualitative ones). Still there are a fifth of the papers in the Special Issue which are psychometric studies, in which usually cultural validations of diagnostic tools have been undertaken, such as the Spanish and Colombian Mobile Phone Problematic Use Scale for Adolescents (MPPUSA [13]), or the Problematic Pornography Consumption Scale (PPCS [37]), which shows the interest to scientifically validate existing measures on problematic use of smartphones, gaming and pornography across cultures [51,52]. This long tradition in psychometrics in the field is probably due to the global phenomena related to the addictive uses of technologies world-wide, and the cross-cultural interest in these common problems in young populations [53]. However, it has also been criticized, as it is quite extensive in comparison with theoretical developments and conceptualisation of these problems and seems not to consolidate enough the field for its recognition as health problems [54]. 

Regarding the method, over half (59%) of the papers published here were correlational studies looking for associations between these problems and internal or external variables. The novelty is these studies is the inclusion of more contextual or environmental factors related to these technological use problems [31,34] and sometimes were treated as predictive ones (i.e., Relational-predictive studies) using predictive aims through statistical techniques to for example extract the factors which seems to cause an outcome (20% of the papers). Only a few studies have real independent variables manipulated by the researchers to cause an effect in other dependent ones, such as new health and educational interventions studied (e.g., Solution-Focused Group Counselling Intervention [25], an app based on cognitive-behavioural principles to avoid smartphone distractions [15]). This means applied research in the field is starting to look for explanations outside the intra-individual sphere through more controlled research designs, through new clinical and educational interventions to promote preventive strategies to minimize harms. Indeed, it can support provisional solutions and support controlling the incidence, and growth of these problems.

Thus, other external variables, such as inter-individual and environmental factors seem to impact Internet use-related addiction problems as intra-individual factors (e.g., sociodemographic, and psychological characteristics). The external indicators which emerged in this Special Issue are related to the Matthew effect (i.e., good premorbid psychosocial adjustment [17]), parent’s education or socio-economic status (i.e., mother´s education [27]), parental care and monitoring (i.e., overprotection promotes these problems [31]), environmental factors (e.g., Internet cafés, advertising [40]), or professional communities (e.g., who manages those affected and their families [22]). Indeed, these contextual factors should also be considered in treatment plans, preventive actions, and policy options [46].

In the first wave of the pandemic in 2020, the lockdowns, restrictions, and isolation have affected individuals’ engagement in addictive behaviours [50,55]. Indeed, the problems which depend on the Internet (the medium frequently used in pandemic times) and the need to be interconnected seems to have produced a growth in these problems [50], which means environmental factors, such as a COVID-19, can have a negative effect on these types of addictive problems in some population groups. However, the clinical, academic, and scientific communities have immediately reacted through prevention alerts and strategies [46,56,57,58,59]. For instance, according to Montag and Elhai [59], who have discussed technology overuse in young populations during the pandemic through Affective Neuroscience Theory, the neglected indirect media effects should not be overseen (i.e., those brain functions involving positive or negative emotions, such as play or sadness systems have been impacted by COVID-19). During the first lockdowns, children have translated their usual diverse leisure activities to screen time activities at home; it seems the problem has been most parents needed to telework and children, apart from home-schooling online, also spent more time online without a parent or adult guidance, which has caused in some cases lack of care, the increment of sadness, and even anger (i.e., disbalance in primary emotional systems). Thus, again, research on the causality of Internet use-related addiction problems (or Internet use disorders [60]) is needed going beyond descriptive and relational quantitative studies which have occupied almost all the scientific body of this field. 

Nevertheless, there are some limitations in the present study. It is based on a limited selection of papers which have been accepted for this Second Special issue based on the editor, the journal academic editorial, and reviewer team through a procedure which usually involved at least two rounds and three reviewers per paper, and a few did not pass the quality check to proceed with the peer-review process. The bias, therefore, in the selection process of the Spanish nationality of the editor an Asiatic journal such as IJERPH, which potentially attracted more proposals from these countries, although an effort has been made to disseminate the invitation world-wide and prioritise locations, topics, and methods not commonly used in this research field to receive a representative pool of a diverse qualified papers. Furthermore, another bias of this editorial rapid review paper is the inherent weakness of conducting a fast review by an author in the same year in which we closed the Special Issue, even with the fact it was extended a few months due to the first wave of COVID-19. However, the IJERPH editorial team and I agreed on this decision and thought it was worth doing this rapid evidence assessment of the papers reviewed, collected, and published in this Special Issue, as it was more successful than initially planned, even with the pandemic factor. 

Interestingly, the field is changing and moved to the need to understand the causes of these specific online problems, and the need to intervene and prevent them from individual and environmental perspectives. In the findings, a limited number of theoretical papers have been included, which are those which study the problems in-depth and sustain empirical research, which is a weakness of the field now highlighted during COVID-19 studies [58,59]. Finally, although the first wave of this pandemic and unprecedented situation has also impacted on the delay of finalising the Special Issue, the number of papers finally published are those which started the review process before March 2020, which probably means health care and scientific production even first impacted by the COVID-19 have also continued providing and producing quality outcomes such as this Special Issue.

## 5. Conclusions

This second Special Issue on “Internet and Smartphone Use-Related Addiction Health Problems: Treatment, Education and Research” has successfully included 40 papers (including this one) from around the globe (except Africa). It includes mainly empirical studies which are relational and a few other types about the adaptation of measures to diagnose these problems, and a few new interventions or preventive strategies to tackle the spectrum of these online usage problems in pre-COVID-19 times. Most of the related issues associated with technological usage problems still seem to be related to individual characteristics of users, who tend to be adults. The problems are about specific online activities and not only focused on gaming disorder. The components which impact their development seem to also be contextual factors, such as the pandemic which seems to have incremented the priorities on researching these health problems from a causal perspective, including environmental factors and intervention and preventive strategies. Thus, although advances have spread around the globe and in diverse Internet use-related addiction problems, maturity of the field still is compromised because there is a need for more qualitative and theoretical research, clinical trials, experimental studies, follow up studies, testing intervention and recovery strategies, policy options, and preventive actions to minimise harms and enhance users’ wellbeing and quality of life, even during present pandemic times of mobility restrictions and more online time consumption.

## Figures and Tables

**Table 1:** Articles included in the Special Issue according to authors’ names, locations, setting and sector, method, problem(s), and findings.

Authors’ Names and Reference	Authors’ Location (Continent(s))	Setting (Data Collection) and Sector (Aim)	Method, Type of Study with Population, and Time (Data Collection)	Problem(s) Studied Regarding the ICT and Other Variables	Main Findings
Lutz Wartberg, and Rudolf Kammerl [11]	Germany (Europe)	Education and health	Empirical and Relational study among problem uses in adolescent-parent dyads, in 2019.	Problematic alcohol use, Problematic Internet use (i.e., Internet addiction (IA): i.e., video game addiction (GD) or social media addiction (SMA)), and mental health	Antisocial behaviour was related to all problematic behaviours. Emotional distress, self-esteem, and hyperactivity/inattention were related to substance-unrelated problematic behaviour. Anger control was related to problematic alcohol use and problematic gaming.
Arnold Alejandro Tafur-Mendoza, Julio César Acosta-Prado, Rodrigo Arturo Zárate-Torres, and Duván Emilio Ramírez-Ospina [12]	Perú and Colombia (America)	Education and health	Empirical and Psychometric study about IA in Peruvian university students	Internet addiction test (IAT) adapted to Spanish Peruvian to measure IA.	The Peruvian IAT has been validated with two factors: time/control and stress/compensate. IAT correlated with daily time of use and social skills.
Verónica Marín-Díaz, Juan Manuel Muñoz-González, and Begoña-Esther Sampedro-Requena [13]	Spain (Europe)	Education and health	Empirical and Psychometric-relational study about problematic smartphone use (PSU) in Spanish and Colombian university students in 2017–2018	PSU through the Mobile Phone Problematic Use Scale for Adolescents (MPPUSA)	The MPPUSA was validated with a model of six factors: tolerance, escape route, disconnection, anxiety, negative consequences, social motivations. However, can only be used with Colombian students, young Spanish women, and students in Social Sciences, with those who had problematic behaviour with the devices, as the ones from Health Sciences students did not have the problem.
Wei Hong, Ru-De Liu, Yi Ding, Rui Zhen, Ronghuan Jiang, and Xinchen Fu [14]	China-United States of America (USA) (Asia and America)	Education and health	Empirical and Relational study between autonomy need dissatisfaction and problematic mobile phone use (PMPU) in Chinese high school’ students.	PMPU, Mobile gaming, autonomy need dissatisfaction, and boredom proneness	Autonomy need dissatisfaction predicted PMPU. Mediators between both were boredom proneness and mobile phone gaming. Thus, a specific psychological need of the mobile phone was detected.
Melina A. Throuvala, Mark D. Griffiths, Mike Rennoldson, and Daria J. Kuss [15]	United Kingdom (UK) (Europe)	Education and Education-Health	Empirical and Predictive study to reduce smartphone uses through a treatment (i.e., an app based on cognitive-behavioural principles to reduce distraction and other psychological outcomes) in undergraduates.	Smartphone use, distraction, nomophobia (NoMO), fear of missing out (FoMO), problem social media use (PSMU), online vigilance, and psychological outcomes	The treatment was effective in reducing smartphone use and distraction, plus other outcomes which were reduced (i.e., impulsivity, stress, anxiety, deficient self-regulation, FoMO, and PSMU) or increased (i.e., self-awareness, mindful attention, self-efficacy), but no effect was observed in PSMU, habitual use or NoMO.
Jui-Kang Tsai, Wei-Hsin Lu, Ray C. Hsiao, Huei-Fan Hu, and Cheng-Fang Yen [16]	Taiwan-USA (Asia and America)	Education and Health	Empirical, and Predictive-relational study about the effect of difficulty in emotion regulation on the occurrence and remission of IA among Taiwanese college students	IA and difficulties in emotion regulation aspects (e.g., impulse control difficulties, lack of emotional awareness, limited access to emotion regulation strategies)	The impulse control difficulties on the difficulties in emotion regulation predicted the incidence of IA during the follow-up period of a year in male participants, but no other aspects predicted the remission of IA. IA did not predict difficulties in emotion regulation.
Seung-Yup Lee, Hae Kook Lee, Jung-Seok Choi, Soo-young Bang, Min-Hyeon Park, Kyu-In Jung, and Yong-Sil Kweon [17]	Korea (Asia)	Health and Health	Empirical, and Longitudinal study (6 months) about the PSU course in South Korean children and adolescents	PSU and mental health indicators	Persistent PSU individuals displayed higher baseline PSU severity and mental health problems at the follow-up. A depressive or anxiety baseline did not influence PSU course. PSU behaved as an addictive disorder. Matthew effect appears in the PSU recovery (i.e., better premorbid psychosocial adjustment prevents it, e.g., more conversations with mothers, perceived happiness, self-esteem)
Montserrat Peris, Usue de la Barrera, Konstanze Schoeps, and Inmaculada Montoya-Castilla [18]	Spain (Europe)	Health and Education	Empirical, and Predictive-relational study about how body self-esteem, personality traits, and demographic factors predict adolescents’ addictive use of social media and the Internet in high school students	Social networking and IA (i.e., IA symptoms, social media use, geek behaviour, nomophobia), and body self-esteem & personality	Four types of adolescents’ IA and as predictors: gender and disinhibition; gender with attractiveness explained social media use; narcissism and neuroticism predict geek behaviour; narcissism best explained nomophobia.
Michelle Colder Carras, Matthew Carras, and Alain B. Labrique [19]	USA (America)	Social and Health	Empirical, and Descriptive mixed-methods study about the techniques to promote healthy play and prevent GD in adult gamers.	GD	Potential targets are specific types of social (e.g., play with others in a group) or self-regulation processes (e.g., set timers or alarms); clear break points and short missions, but loot boxes were not mentioned.
María Luisa Ballestar-Tarín, Conchín Simó-Sanz, Elena Chover-Sierra, Carlos Saus-Ortega, Carmen Casal-Angulo, and Antonio Martínez-Sabater [20]	Spain (Europe)	Health and Education	Empirical and Psychometric-relational study about PSU in Spanish in university students in 2017.	PSU and sociodemographic variables. The Smartphone Addiction Inventory adapted to Spanish (SPAI-Spanish) with a cut-off point.	The SPAI-Spanish has been validated with sociodemographic differences. The cut-off was established for those on perceived risk of PSU.
Siti Rubiaehtul Hassim, Wan Nor Arifin, Yee Cheng Kueh, and Nor Azwany Yaacob [21]	Malasya (Asia)	Education and Health	Empirical and Psychometric-relational study about PSU in Malay medical students in 2017.	PSU, IA, and sociodemographic variables. The Smartphone Addiction Scale adapted to Malay (SAS-M).	The SAS-M has been validated which correlated with IA. The cut-off was established for those on perceived risk of PSU
Darryl Mead and Mary Sharpe [22]	UK (Europe)	Health	Theoretical, Descriptive study about the need of recognition of the problematic usage of pornography (PUP) within a European Manifesto.	PUP	The Manifesto for a European research network into Problematic Usage of the Internet in 2018 did not covered the priority of the PUP as other IA problems (e.g., community-focused issues, professional communities affected).
Young-Mi Ko, Sungwon Roh, and Tae Kyung Lee [23]	South Korea (Asia)	Social and Health	Empirical, Predictive-relational study between problematic Internet shopping (PIS) and dissociative experiences in adults.	PIS and sociodemographic characteristics, alcohol use, caffeine intake, online shopping behaviours, and Psychopathological assessments	The risk of PIS was related to an increased tendency toward dissociation and impulsivity. PIS with dissociation have more stress, gambling problems, and impulsivity.
Sarah E. Domoff, Emma Q. Sutherland, Sonja Yokum, and Ashley N. Gearhardt [24]	USA (America)	Education and Health	Empirical, Relational study between PSU and eating behaviours in adolescents between 2015 and 2017.	PSU and emotion regulation difficulties, impulsivity, maladaptive eating behaviours, and adiposity	PSU is associated with these difficulties, dysregulated eating, restrained eating, food addiction, and higher percent body fat; being the difficulties a mediator.
Xinhe Zhang, Xiaoxuan Shi, Shuowei Xu, Jingwen Qiu, Ofir Turel, and Qinghua He [25]	China-USA (Asia and Ameria)	Education and Health	Empirical, Predictive experimental study on IA in Chinese university students in 2018.	To test a treatment for IA (i.e., a 5-week solution-focused group counselling)	The treatment was effective even during a follow up, especially in compulsive-withdrawal and tolerance symptoms.
Sina Ostendorf, Elisa Wegmann, and Matthias Brand [26]	Germany (Europe)	Education and Health	Empirical, Relational study between social-networks-use disorder (SNUD) risks (need to belong, NTB), and protective (online self-regulative competences, OSRC) factors in adolescents during 2017.	SNUD (SMA) and NTB, OSRC with sociodemographic variables	Highest SNUD with high NTB and low OSRC, especially when older. The importance of improving specific competences to prevent SNUD
Adnan Veysel Ertemel, and Ela Ari [27]	Turkey (Asia)	Education and Health	Empirical, Predictive study on PSU in adolescent students in 2018	To test a PSU educative program (i.e., consumer behaviour theories and unhook and gamification techniques)	The PSU significantly decreased after the program, depending on gender, mother’s education, and class levels.
Jinhee Lee, Joung-Sook Ahn, Seongho Min, and Min-Hyuk Kim [28]	Korea (Asia)	Education and Health	Empirical, Relational study between content type smartphone use and psychological characteristics, addiction propensity, time of its use and PSU in adolescents during 2017.	PSU, Smartphone uses (study, social networking sites (SNS), game, entertainment), Psychology (depressive mood, suicidal ideation),	Psychological characteristics were associated with SNS use, which showed higher addiction (overuse & adverse consequences).
Élodie Verseillié, Stéphanie Laconi, and Henri Chabrol [29]	France (Europe)	Education and Health	Empirical, Relational-predictive study between psychopathology and problematic SNS uses (or SMA) in adults.	SMA (Facebook and Twitter), and psychopathology (personality traits, depression, anxiety symptoms, stress)	Problem SNS uses were predicted by personality traits (depressive and anxiety), only stress predicted problematic Facebook use.
Hiu Yan Wong, Hoi Yi Mo, Marc N. Potenza, Mung Ni Monica Chan, Wai Man Lau, Tsz Kwan Chui, Amir H. Pakpour, and Chung-Ying Lin [30]	Hong Kong, USA, Iran, Sweden (Asia, America and Europe)	Education and Health	Empirical, Relational study between Internet gaming disorder (IGD), SMA, sleep quality, psychological distress among Hong Kong young adults in 2019.	IGD, SMA and distress (depression, anxiety, and stress)	IGD is associated with distress, and a bit with poor sleep quality. SMA similarly associated with distress, but higher with poorer sleep quality.
Anna Faltýnková, Lukas Blinka, Anna Ševčíková, and Daniela Husarova [31]	Czech Republic, Slovakia (Europe)	Education and Health	Empirical, Relational-predictive study between IA, and family environment in Slovakian adolescents.	IA and family type, economic status, parental care, parental control, parental monitoring, communication, and time spent.	Higher parental care and parental monitoring predicted lower IA, while higher parental overprotection and lower socioeconomic status predicted higher IA.
Marta Beranuy, Juan M. Machimbarrena, M. Asunción Vega-Osés, Xavier Carbonell, Mark D. Griffiths, Halley M. Pontes, and Joaquín González-Cabrera [32]	Spain (Europe)	Education and Health	Empirical and Psychometric-relational study about IGD short form scale (IGDS9-SF) in Spanish and its relationship with online gambling (OG) and quality life (QoL) in young students in 2019.	To adapt and validate to Spanish the IGDS9-SF, and to relate it to PSU, OG	The Spanish IGDS9-SF was valid and reliable, 1.9% of gamers had IGD. IGD was positively related to PSU and OG. Those with IGD has less QoL.
Vega González-Bueso, Juan José Santamaría, Ignasi Oliveras, Daniel Fernández, Elena Montero, Marta Baño, Susana Jiménez-Murcia, Amparo del Pino-Gutiérrez, and Joan Ribas [33]	Spain (Europe)	Health and Health	Empirical and Descriptive study to classify IGD adolescent patients according to their personality & describe them clinically and demographically.	IGD	There were 2 types: I “higher comorbid symptoms” (introversive, inhibited, doleful, unruly, forceful, oppositional, self-demeaning, borderline) & type II “lower comorbid symptoms” (histrionic, egotistic and conforming)
Xinchen Fu, Jingxuan Liu, Ru-De Liu, Yi Ding, Jia Wang, Rui Zhen, and Fangkai Jin [34]	China and USA (Asia and America)	Education and Health	Empirical, Relational-predictive study between PSU, and parental monitoring in Chinese adolescents	PSU	Parental monitoring predicted PSU, children’s escape motivation mediated it, while shyness moderated the path and the impact of monitor PSU
Ningyuan Guo, Tzu Tsun Luk, Sai Yin Ho, Jung Jae Lee, Chen Shen, John Oliffe, Sophia Siu-Chee Chan, Tai Hing Lam, and Man Ping Wang [35]	China and Canada (Asia and America)	Education and Health	Empirical, Relational-predictive study between PSU, and anxiety, depression, and mental well-being in Hong Kong Chinese adults in 2017.	PSU & psychological variables (depression, anxiety, subjective happiness, and mental well-being)	PSU was associated with higher anxiety and depression, and lower happiness and wellbeing)
Beifang Fan, Wanxing Wang, Tian Wang, Bo Xie, Huimin Zhang, Yuhua Liao, Ciyong Lu, and Lan Guo [36]	China (Asia)	Education and Health	Empirical, Relational-predictive study between IA, and non-medical use of prescription drugs (NMUPD), and depression in Chinese adults, 2017.	IA and NMUPD (use of opioid, sedative), and depression	IA associated with depression and frequent use of opioid and sedative drugs, elevating the risk of this addiction.
Lijun Chen, and Xiaoliu Jiang [37]	China (Asia)	Education and Health	Empirical, Psychometric mixed methods study about the online PUP or problematic Internet pornography use (IPU) in Chinese adults.	PUP or IPU	The Problematic Pornography Consumption Scale (PPCS) had stronger reliability and validity, including criterion validity, greater sensitivity, and acceptable specificity.
Ki Hyeon Kwak, Hyun Chan Hwang, Sun Mi Kim, and Doug Hyun Han [38]	Korea (Asia)	Health and Health	Empirical, Relational study between IGD, psychology, brain activity between pro-gamers and IGD Korean adolescents between 2016 and 2017.	IGD, psychology (depressed mood, anxiety), and brain activity (resting-state functional magnetic resonance imaging)	Both groups displayed increased brain activity in the parietal lobe (attention), but IGD adolescents showed higher brain activity within the left orbitofrontal cortex (impulsivity and aggression).
Bernadeta Lelonek-Kuleta, Rafał P. Bartczuk, Michał Wiechetek, Joanna Chwaszcz, and Iwona Niewiadomska [39]	Poland (Europe)	Education and Health	Empirical, Relation-predictive study about aspects of gambling among Polish adults.	e-Gambling (OG) and its activities	4.1% did e-gambling (lotteries, sports betting), 26.8% classed as problem gamblers. Men, younger, who earnt less were more often involved in e-gambling.
Sulki Chung, Jaekyoung Lee, and Hae Kook Lee [40]	Korea (Asia)	Education and Health	Empirical, Relation study about personal, family/school, Internet use, and environmental factors among Korean adolescents in 2015.	IA with individual (psychology, family cohesion, attitudes toward academic activities) and environmental factors (Internet characteristics, accessibility to PC cafés, and exposure to Internet game advertising).	6% IA: using the Internet earlier; had higher levels of depression, compulsivity, aggressiveness, lower family cohesion; higher accessibility to PC cafés and exposure to Internet game advertising. Environmental factors had a greater influence than family or school-related factors
Amandine Luquiens, Aline Dugravot, Henri Panjo, Amine Benyamina, Stéphane Gaïffas, and Emmanuel Bacry [41]	France (Europe)	Social and Health	Empirical, Predictive study about the effect of self-exclusion in online poker gambling as compared to matched controls, after the end of the self-exclusion period with French adult gamblers in 2016.	OG	Effects of self-exclusion and short-duration self-exclusion were found for money and time spent over a year. Short-duration self-exclusions showed no effect on the heavy gamblers.
Myungsuh Lim [42]	Korea (Asia)	Social and Health	Empirical, Relational study about the effect of Facebook addiction and other demographic and psychological characteristics with USA adults.	SMA (Facebook), and demographics (age and gender), social exclusion, surveillance use, and narcissistic grandiosity	Social exclusion and surveillance were associated with SMA.
Kuan-Ying Hsieh, Ray C. Hsiao, Yi-Hsin Yang, Kun-Hua Lee, and Cheng-Fang Yen [43]	Taiwan and USA (Asia and America)	Education and Health	Empirical, Relational study about self-identity confusion and IA and the mediating effects of psychological inflexibility and experiential avoidance (PI/EA) indicators in Taiwanese college students.	IA, self-identity (Self-Concept and Identity Measure), PI/EA (Acceptance and Action).	The severity of self-identity confusion was associated with both the severity of PI/EA and IA. The severity of PI/EA indicators was associated with IA.
Ju-Yu Yen, Huang-Chi Lin, Wei-Po Chou, Tai-Ling Liu, and Chih-Hung Ko [44]	Taiwan (Asia)	Health and Health	Empirical, Relational-predictive study about resilience, perceived stress, depression, and IGD in Taiwanese young adults in 2013.	IGD and psychological variables.	IGD group had a lower resilience, higher stress, and depression than the control group. Low resilience was associated with a higher IGD and depression. Depression was more associated with IGD than resilience. Depression and stress coping interventions should be provided for those with IGD with low resilience or high stress.
Yi-Ping Hsieh, Cheng-Fang Yen, and Wen-Jiun Chou [45]	Taiwan and USA (Asia and America)	Health & Health	Empirical, Psychometric mixed methods study about the Parental Smartphone Use Management Scale (PSUMS) and relationships with PSU and attention deficit hyperactivity disorder (ADHD) in Taiwanese parents of adolescents with ADHD in 2014–2015.	PSU and ADHD	The PSUMS had good factorials validity and high reliabilities. Parents of children with smartphone addiction yielded lower scores on all three PSUMS subscales (i.e., reactive management, proactive management, and monitoring) than parents of children without smartphone addiction
Olatz Lopez-Fernandez, and Daria J. Kuss [46]	Australia and UK (Oceania and Europe)	Health	Theoretical, literature review on Internet use-related addiction problems on a European scale	IA, IGD, OG	Those with problematic uses are educated male adolescents, with comorbid disorders, being gaming and gambling the most severe addictions. Cognitive behavioural therapy was the main treatment. Policy options and preventive strategies are proposed for public health in Europe.
Antonio-José Moreno-Guerrero, Gerardo Gómez-García, Jesús López-Belmonte, and Carmen Rodríguez-Jiménez [47]	Spain (Europe)	Health	Theoretical, literature review on IA in the web of science database	IA	The evolution in the study of the IA is constant, articles in English, in psychiatry, Prof Griffiths, from Nottingham Trent University, publishing in *Computers in Human Behavior* and the *Journal of Behavioral Addictions*, and USA has greater interest in production.
Sheila Yu, and Steve Sussman [48]	USA (America)	Health	Theoretical, systematic review on measures of smartphone addiction (SA) and PSU to check if they are distinct from other addictions, and how may fall on a continuum of addictive behaviors	PSU and SA related to other technological addictions	Most studies neither distinguished SA from other technological addictions nor clarified whether SA was an addiction to the actual smartphone device or its features. No theory to explain the etiologic origins or causal pathways of SA and its associations.
Christian Montag, Bernd Lachmann, Marc Herrlich, and Katharina Zweig [49]	Germany (Europe)	Health	Theoretical, review to analyze on several prominent smartphone apps to carve out over-usage smartphone apps	PSU apps (social media apps and Freemium games)	These app-elements are linked to classic psychological/economic theories, e.g., mere-exposure, endowment, and Zeigarnik effects, but also to psychological mechanisms triggering social comparison.

Note: the acronyms used are the following: Internet addiction (IA), (video) gaming disorder (GD), social media addiction (SMA), Internet addiction test (IAT), problematic smartphone use (PSU), Mobile Phone Problematic Use Scale for Adolescents (MPPUSA), problematic mobile phone use (PMPU), nomophobia (NoMO), fear of missing out (FoMO), problem social media use (PSMU), Smartphone Addiction Inventory adapted to Spanish (SPAI-Spanish), Smartphone Addiction Scale adapted to Malay (SAS-M), problematic usage of pornography (PUP), problematic Internet shopping (PIS), social-networks-use disorder (SNUD), need to belong (NTB), online self-regulative competences (OSRC), social networking sites (SNS), Internet gaming disorder (IGD), IGD short form scale (IGDS9-SF), online gambling (OG), quality life (QoL), non-medical use of prescription drugs (NMUPD), Internet pornography use (IPU), psychological inflexibility (PI), experiential avoidance (EA), Parental Smartphone Use Management Scale (PSUMS), attention deficit hyperactivity disorder (ADHD), smartphone addiction (SA).

## Data Availability

The data presented in this study are openly available at https://www.mdpi.com/journal/ijerph/special_issues/Internet_Smartphone_Addiction.
